# Supplementary material for: Comprehensive Transcriptome Reveals an Opposite Regulatory Effect of Plant Growth Retardants in Controlling Seedling Overgrowth between Roots and Shoots
Source: Int J Mol Sci. 2019 Jul 5;20(13):3307. doi: 10.3390/ijms20133307 (PMC6650903; doi:10.3390/ijms20133307)
Supplement: Supplementary file 1 [file ijms-20-03307-s001.zip › ijms-522547 sp for proof/ijms-522547 suppl legends.pdf]

Figure S1. Comparison of transcriptome data under CCC and BPBZ treatment in tomato shoots and roots. The scatter plots showed a good quality of transcriptome datasets derived by CCC and PBZ treatment both in shoots and roots.

Figure S2. Phylogenetic analysis of genome-wide *SlEXPs* genes in tomato. The genome-wide 40 expansin genes in tomato were clustered in the phylogenetic tree, and this was constructed in MEGA 6.0 software with the Neighbor-Joining method. The bootstrap analysis was performed with 1000 iterations.

Figure S3. The opposite regulatory mechanism between roots and shoots induced by plant growth retardants in controlling seedling overgrowth. The plant growth retardants CCC and PBZ decrease the endogenous GA content of tomato seedlings both in the aboveground shoot and the underground root, then induce an opposite regulation between shoots and in roots, which results in a significant restriction in the shoot but a horizontal elongation in the root.

Table S1. Morphological statistical analysis of tomato seedlings under the conditions of CK, CCC, and PBZ.

Table S2. Efficiency and R2 values (coefficients of determination) of primer pairs.

Table S3. Significant DEGs involved in tomato shoots and roots under CCC/PBZ treatment.

Table S4. G-box analysis of tomato EXPs promoters.
