# Supplementary material for: Comprehensive Transcriptome Reveals an Opposite Regulatory Effect of Plant Growth Retardants in Controlling Seedling Overgrowth between Roots and Shoots
Source: Int J Mol Sci. 2019 Jul 5;20(13):3307. doi: 10.3390/ijms20133307 (PMC6650903; doi:10.3390/ijms20133307)
Supplement: Supplementary file 1 [file ijms-20-03307-s001.zip › ijms-522547 sp for proof/Supplementary Files/Supplementary Table 1.pdf]

Supplementary Table 1. Morphological statistical analysis of tomato seedling under the condition of CK, CCC and PBZ

Stage	Treatment	Hypocotyl (cm)	Plant height (cm)	Stem diameter (mm)	Root length (cm)
**Two-leaf stage**	CK	5.06±0.52	8±0.98	1.98±0.32	17.13±1.52
	CCC	2.35±0.22**	2.68±0.32**	1.36±0.12**	4.69±0.57**
	PBZ	2.09±0.15**	2.58±0.28**	1.21±0.23**	10.02±1.02**
**Three-leaf stage**	CK	5.45±0.63	9.4±0.76	2.77±0.32	17.35±1.61
	CCC	2.41±0.32**	3.35±0.47**	1.65±0.15**	4.95±0.62**
	PBZ	2.11±0.28**	2.95±0.49**	1.74±0.12**	10.1±1.23**
**Four-leaf stage**	CK	5.61±0.54	18.27±1.5	4.41±0.54	18.55±1.82
	CCC	2.51±0.34**	3.57±0.58**	2.01±0.25**	8.45±0.92**
	PBZ	2.18±0.3**	3.72±0.42**	2.24±0.21**	11±1.32**

*P < 0.05, **P < 0.01
